# Autofluorescence Quenching in Decellularized Plant Scaffolds for Tissue Engineering

**DOI:** 10.1007/s10439-025-03829-5

**Published:** 2025-08-16

**Authors:** Nick Merna

**Affiliations:** Bioengineering Program, Fred DeMatteis School of Engineering and Applied Science, Hofstra University, 228 Science and Innovation Center, Hempstead, NY 11549 USA

**Keywords:** Autofluorescence quenching, Decellularized scaffolds, Plant-based tissue engineering

## Abstract

**Purpose:**

Autofluorescence in plant-derived scaffolds interferes with fluorescence imaging by overlapping with commonly used fluorophores such as Hoechst and FITC. This limits the ability to visualize cell behavior and scaffold integration in tissue engineering applications. This study evaluated whether copper sulfate, ammonium chloride, or sodium borohydride can reduce autofluorescence in decellularized plant scaffolds without compromising mechanical integrity or cell viability.

**Methods:**

The effectiveness of the three quenching agents was evaluated in decellularized leatherleaf viburnum, spinach, and parsley scaffolds. Spectral scans were used to characterize baseline autofluorescence. Treated and untreated scaffolds were imaged in Hoechst, FITC, and 633 nm channels. Autofluorescence intensity, quenching stability over 24 h, mechanical properties, and endothelial cell viability were assessed. Imaging of cell seeded scaffolds evaluated improvements in visualization after treatment.

**Results:**

Spectral scans revealed strong autofluorescence in the blue and green channels, overlapping with Hoechst and FITC. Copper sulfate reduced autofluorescence more effectively than ammonium chloride or sodium borohydride and improved nuclear visualization, with consistent performance across scaffold types. However, endothelial cell viability declined in copper-treated leatherleaf and parsley scaffolds but remained high in spinach. No significant changes in tensile strength or elastic modulus were observed after treatment.

**Conclusion:**

Copper sulfate is a highly effective and stable quenching agent for reducing autofluorescence in plant-derived scaffolds. While suitable for post-fixation imaging, scaffold-specific effects on viability limit its use in live-cell applications. Autofluorescence reduction was achieved without compromising scaffold mechanics. Ammonium chloride and sodium borohydride may be preferable when preserving cell viability is a priority.

## Introduction

Small-diameter vascular grafts face significant challenges due to thrombosis, intimal hyperplasia, and poor long-term patency, limiting their clinical success. These issues often arise due to the inability of synthetic grafts to mimic the dynamic biological and mechanical properties of native blood vessels [1, 2]. Decellularized scaffolds address these limitations by preserving the native extracellular matrix architecture while reducing immunogenicity [3–5], making them promising candidates for vascular graft applications. Recently, plant-derived scaffolds such as leatherleaf viburnum, spinach, and parsley have emerged as cost-effective and sustainable alternatives, offering vascular-like structures suitable for graft design [3, 6]. However, the inherent autofluorescence of plant-derived scaffolds, caused by lignin, chlorophyll, and polyphenolic molecules, overlaps with emission spectra of fluorophores such as Hoechst (405 nm) and FITC (488 nm), reducing signal-to-noise ratio and obscuring labeled structures [7–9]. It interferes with visualization of cellular activity and scaffold-cell interactions, which are critical for evaluating tissue-engineered constructs [10, 11]. Addressing autofluorescence is essential for applying plant-derived scaffolds in regenerative medicine, particularly for imaging-intensive studies that rely on fluorescent markers to track cell behavior and scaffold integration.

Despite the importance of addressing autofluorescence, there is a lack of standardized quenching protocols specifically tailored for plant-based scaffolds. Quenching agents reduce autofluorescence by chemically modifying or suppressing fluorescent signals. While quenching agents such as copper sulfate (CS), ammonium chloride (AC), and sodium borohydride (SB) have been widely used in fixed mammalian tissue studies, their effectiveness depends on the chemical nature of the underlying autofluorescent compounds [12]. In plant-derived scaffolds, autofluorescence is primarily driven by lignin and polyphenols, rather than aldehyde crosslinks. CS was selected based on its ability to alter the electronic states of chromophores and suppress lipofuscin-associated autofluorescence in neural tissues without affecting specific fluorophores [13, 14]. Given the structural similarity between lipofuscin and plant-derived fluorophores, CS was expected to be effective in this context. AC was selected for its routine use in reducing aldehyde-based fluorescence in formalin-fixed tissues [15, 16], and SB for its ability to chemically reduce aldehydes and ketones to less reactive forms [17, 18]. Because the scaffolds were fixed in formaldehyde during preparation, both AC and SB were also included to determine whether they could suppress fixative-induced background fluorescence in addition to plant-derived signals. Identifying effective quenching agents will improve visualization of scaffold-cell interactions, enabling better evaluation of plant-derived scaffolds in regenerative medicine applications such as small-diameter vascular grafts [5, 19].

This study aims to address this challenge by evaluating the effectiveness of CS, AC, and SB in reducing autofluorescence in decellularized plant scaffolds. The specific objectives of this study are threefold:

1. To evaluate the effectiveness of CS, AC, and SB in reducing autofluorescence in leatherleaf viburnum, spinach, and parsley scaffolds across Hoechst, FITC, and far-red (633 nm) channels.
2. To assess the time-dependent stability of autofluorescence reduction by quenching agents over 24 h.
3. To evaluate the effect of quenching agents on fluorescence imaging clarity for visualizing endothelial cells (EC) and smooth muscle cells (SMC) on scaffolds.

Together, these aims support the development of reliable fluorescence imaging protocols for plant-derived scaffolds. Improved visualization of cellular integration and scaffold remodeling will facilitate broader use of these materials in regenerative medicine, particularly for small-diameter vascular grafts.

## Methods

### Preparation of Decellularized Plant Scaffolds

Fresh leaves from three plant species (leatherleaf viburnum, spinach, and parsley) were decellularized to generate acellular scaffolds for autofluorescence analysis [5]. Leaves were decellularized in 2% sodium dodecyl sulfate (Sigma-Aldrich) for 72 h in deionized water, followed by 6 h in clearing solution (10% bleach and 0.1% Triton X-100 in deionized water). The scaffolds were then fixed in 10% formaldehyde for 10 min at room temperature and washed thoroughly with phosphate-buffered saline (PBS) to remove residual fixative.

### Spectral Scans

Spectral scans were conducted to characterize the autofluorescence of decellularized leatherleaf viburnum scaffolds. This technique captures fluorescence emission across a range of wavelengths for a specific excitation source, providing a detailed profile of autofluorescence or fluorophore emissions. Scans were performed using excitation wavelengths of 405, 488, 561, and 640 nm. At 20 × magnification, images were acquired with emissions ranging from 408 to 688 nm, in 5 nm increments, using a 15 nm bandpass filter to ensure precise spectral resolution.

### Quenching of Autofluorescence

To reduce background autofluorescence in decellularized plant scaffolds, three quenching agents—SB, CS, and AC—were tested at various concentrations and treatment times (*N* = 3).

SB (Sigma-Aldrich) was prepared fresh in deionized water due to its instability in solution. Scaffolds were treated with 0.1 M (low), 0.5 M (medium), or 1 M (high) concentrations for 10 or 20 min at room temperature. Following treatment, leatherleaf scaffolds were thoroughly washed with PBS three times (5 min per wash) to remove residual SB.

CS (Sigma-Aldrich) was prepared at concentrations of 0.01 M (low), 0.05 M (medium), or 0.1 M (high) in deionized water. Leatherleaf scaffolds were incubated in CS solutions for 10 or 20 min at room temperature. After treatment, the scaffolds were washed three times with PBS to remove any unbound copper ions.

For AC treatments (Sigma-Aldrich), solutions were prepared in deionized water at concentrations of 0.02 M (low), 0.1 M (medium), and 0.2 M (high). Leatherleaf scaffolds were incubated in the AC solution for either 10 min or 20 min at room temperature. Following treatment, scaffolds were washed thoroughly in PBS to ensure complete removal of AC.

Untreated scaffolds served as controls for comparison of autofluorescence. All quenching treatments and solution preparations were conducted in a fume hood to ensure safety and proper ventilation, particularly for SB, which releases flammable hydrogen gas when in contact with water. After quenching, scaffolds were imaged and analyzed to evaluate the reduction in autofluorescence relative to untreated samples.

To evaluate the generalizability of autofluorescence quenching protocols, additional experiments were conducted using decellularized spinach and parsley scaffolds. Samples (*N* = 3 per condition) were treated with 0.1 M CS, 0.2 M AC, or 1.0 M SB for 20 min at room temperature. Quenching was performed in deionized water, followed by three 5-min PBS washes. These concentrations and durations were selected based on prior experiments in viburnum that demonstrated effective autofluorescence reduction. Treated scaffolds were imaged within 1 h of quenching.

### Fluorescence Imaging

Fluorescence imaging was performed using an FV3000 scanning confocal microscope (Olympus Corporation, Shinjuku City, Tokyo, Japan) to evaluate the efficacy of each quenching agent, with images captured in Hoechst (nuclear stain), FITC, and 633 nm channels. Exposure time and laser intensity were maintained at a constant value for each channel across all experiments, and imaging was conducted at 20 × magnification. Z-stack imaging was performed for each sample, capturing 25 slices at 1 µm per slice to ensure comprehensive coverage of the scaffold structure. A maximum intensity projection was created from the Z-stack to provide a composite image of the fluorescence signal across the depth of each sample. Untreated scaffolds were included as controls to allow direct comparison of autofluorescence levels with and without quenching treatments.

### Mechanical Testing

Decellularized leatherleaf viburnum, spinach, and parsley scaffolds were cut into 5 × 10 mm strips for tensile testing. Digital calipers were used to measure the gauge length, thickness, and width of each sample. Samples were mounted in a uniaxial tensile tester and pulled at a constant rate of 0.08 mm/s until failure. Load and extension data were recorded, and tensile strength and elastic modulus were calculated from the stress–strain curve. For each scaffold type and treatment group (CS, AC, SB, and untreated), three to five samples were tested in the hydrated state.

### Image Analysis

Quantification of fluorescence intensity was performed using ImageJ on each Z-stack projection. Three regions of interest were defined on each image. The mean fluorescence intensity (mean gray value) for each condition was calculated and compared to the mean intensity from untreated control scaffolds. Additionally, a time-course experiment was conducted to evaluate the stability of the quenching effect over time by measuring autofluorescence at 0, 6, and 24 h post-treatment. This allowed assessment of whether the autofluorescence reduction remained stable, an important factor for applications requiring extended imaging periods.

### Cell Seeding and Imaging

Following initial experiments to assess quenching agents, CS emerged as the most effective for reducing autofluorescence in decellularized plant scaffolds. Thus, to evaluate its performance in cell-seeded scaffolds, primary rat aortic ECs or rat aortic SMCs (CellApplications) were seeded onto the CS-treated scaffolds at passages 5 and 8, respectively. Cells were seeded at a density of 625,000 cells/cm^2^ and incubated for 24 h in growth medium (CellApplications) under standard cell culture conditions to allow for cell adhesion.

After the 24-h incubation, cell-seeded scaffolds were fixed in 10% formaldehyde for 10 min at room temperature to preserve cellular structure. Following fixation, scaffolds were treated with 0.05 M CS for 10 min to quench autofluorescence. The quenched scaffolds were then washed three times in PBS to remove residual CS. Additional cell-seeded scaffolds received no CS as a control.

Samples were then stained with Hoechst to label cell nuclei. Fluorescence imaging was conducted as described above, with images captured in the Hoechst channel using a confocal microscope. Imaging parameters, including exposure time and laser intensity, were held constant across all samples, with 20 × magnification and Z-stacks captured at 1 µm per slice over 25 slices. A maximum intensity projection was created from the Z-stacks to provide a comprehensive image for fluorescence intensity analysis.

To evaluate cytocompatibility of CS, AC, and SB, decellularized leatherleaf, spinach, and parsley were fixed in 10% formaldehyde for 10 min and treated with 0.1 M CS, 1 M SB, or 0.2 AC for 20 min. Untreated scaffolds served as controls. All samples were then rinse in sterile PBS for 48 h before seeding with ECs at 120,000 cells/cm^2^. After 24 h of incubation in growth medium, viability was assessed using a live/dead cell imaging kit (#R37601, Invitrogen). Viable cells were counted, and the ratio of live cells to total cells was calculated as previously described [25].

### Statistical Analysis

Statistical comparisons among groups of three or more were performed using one-way ANOVA. Significant differences were further analyzed with pairwise comparisons using Tukey’s post hoc test, with *p* < 0.05 indicating statistical significance. All analyses were carried out in Microsoft Excel (Redmond, WA, USA), and data are presented as mean ± standard deviation.

## Results

### Autofluorescence and Spectral Profiles of Decellularized Leatherleaf Viburnum

**Fig. 1:**
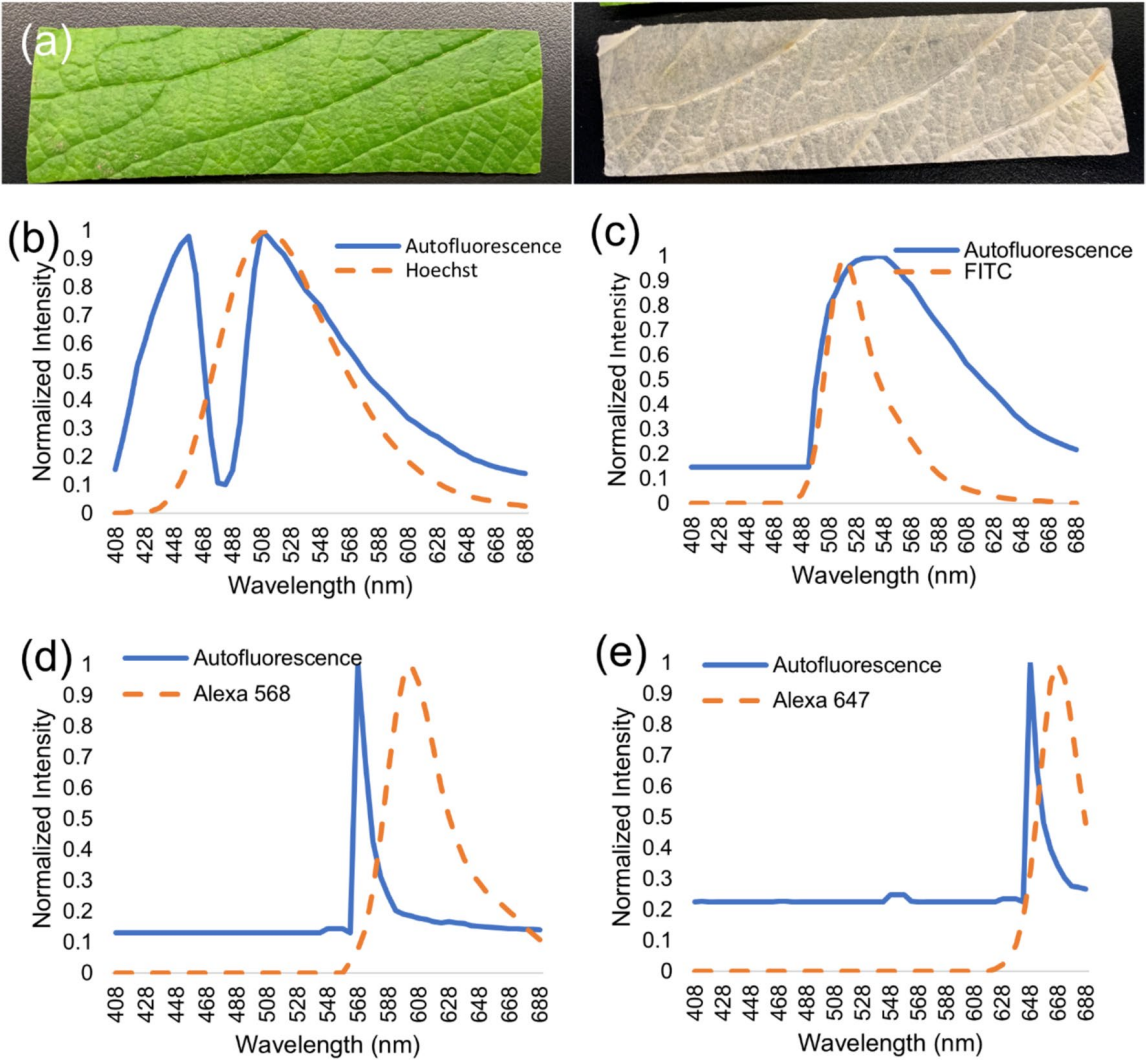
**a** Leatherleaf viburnum samples before and after decellularization. **b–e** Spectral scans at 405, 488, 561, and 640 nm excitation showing overlap between scaffold autofluorescence and emission profiles of common fluorophores

The decellularization process resulted in complete removal of cellular material from the leatherleaf viburnum samples (Fig. 1a), as previously described [5]. Spectral scans of the decellularized samples revealed significant autofluorescence across multiple excitation and emission wavelengths (Fig. 1b–e).

At an excitation wavelength of 405 nm, the emission profile of the decellularized scaffold exhibited substantial overlap with the emission spectrum of Hoechst, indicating potential interference during imaging with this nuclear stain (Fig. 1b). Similarly, excitation at 488 nm resulted in a strong emission overlap with the FITC emission spectrum, suggesting significant autofluorescence interference in this spectral range (Fig. 1c). In contrast, excitation at 561 and 640 nm showed minimal overlap with the emission spectra of Alexa Fluor 568 and Alexa Fluor 647, respectively (Fig. 1d–e). These results highlight that autofluorescence from the decellularized leatherleaf viburnum is more pronounced in the lower wavelength range (405–488 nm) and diminishes at higher wavelengths (561–640 nm), providing insights into potential challenges and opportunities for fluorescence imaging.

### Fluorescence Imaging of Decellularized Leatherleaf Viburnum Scaffolds

**Fig. 2:**
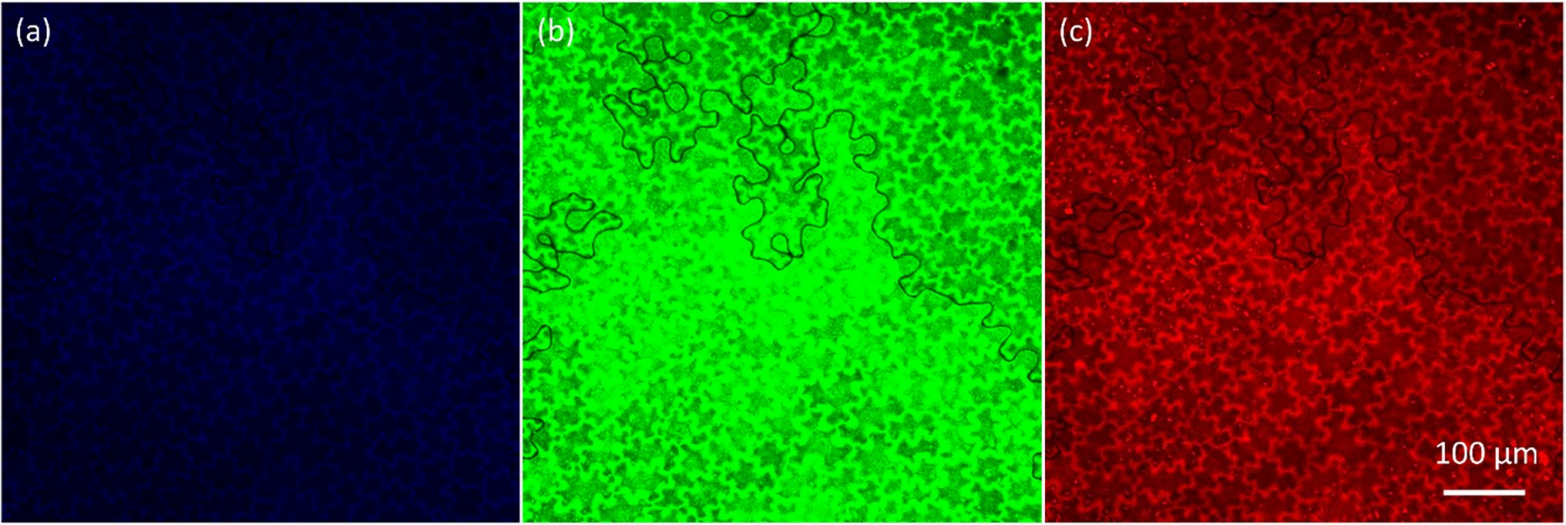
Fluorescence images of acellular, decellularized leatherleaf scaffolds using **a** Hoechst, **b** FITC, and **c** 633 nm channels showing channel-dependent autofluorescence intensity

Fluorescence imaging of acellular, decellularized leatherleaf viburnum scaffolds revealed varying levels of autofluorescence across different channels (Fig. 2). FITC (green) and 633 nm (red) imaging revealed strong autofluorescence, with bright fluorescence emission observed throughout the scaffold. This suggests that autofluorescence may significantly interfere with imaging when using fluorophores emitting in these spectral ranges. In contrast, imaging in the Hoechst (blue) channel demonstrated relatively lower levels of autofluorescence, indicating less potential interference when using Hoechst as a nuclear stain. These findings highlight the importance of effective quenching strategies to minimize autofluorescence, particularly in the green and red emission ranges, to ensure accurate fluorescence-based imaging of the scaffolds.

### Comparison of Quenching Agents for Autofluorescence Reduction

**Fig. 3:**
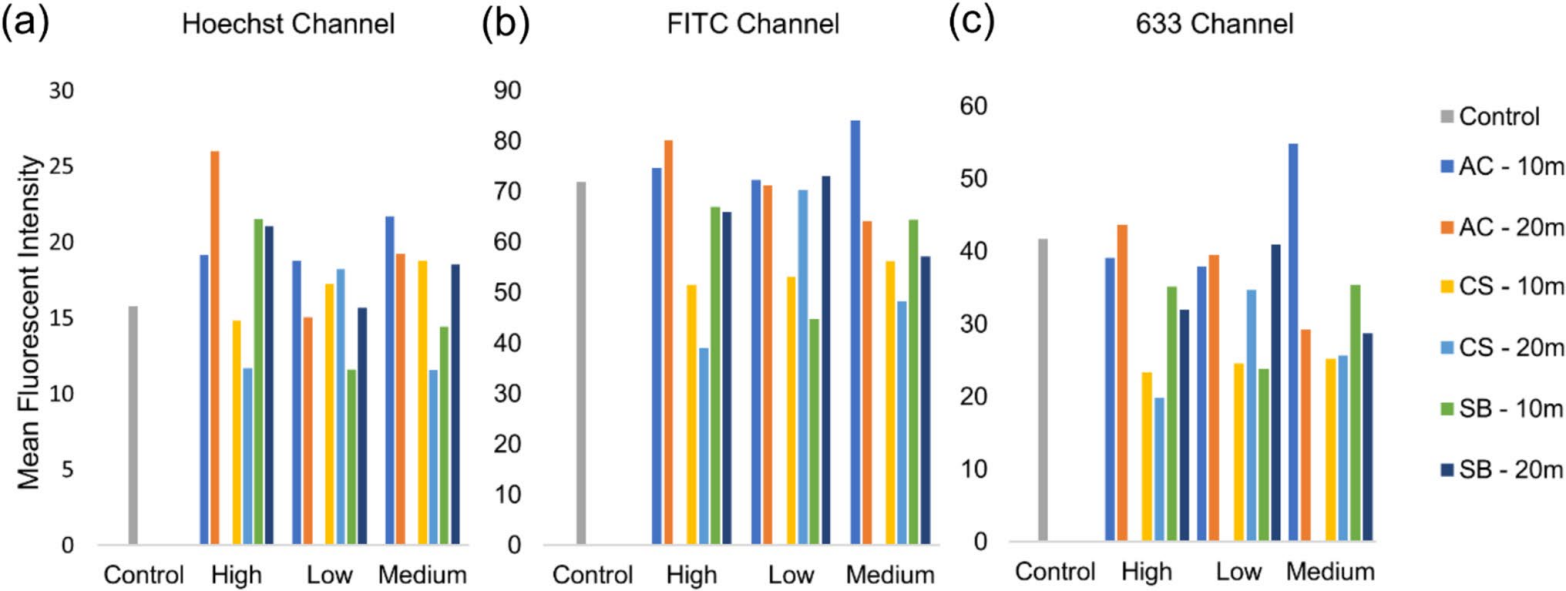
Mean fluorescence intensity in scaffolds treated with copper sulfate, ammonium chloride, or sodium borohydride at varying concentrations and treatment times. **a–c** show results for Hoechst, FITC, and 633 nm channels, respectively

As shown in Fig. 3, the effectiveness of three quenching agents (CS, AC, and SB) was assessed for reducing autofluorescence in decellularized leatherleaf scaffolds across Hoechst, FITC, and far-red (633 nm) channels. Untreated controls exhibited consistently higher fluorescence intensity across all channels, highlighting the baseline autofluorescence of the scaffolds.

In the Hoechst channel, only CS demonstrated a noticeable reduction in autofluorescence. This reduction was most pronounced (26%) at medium and high concentrations, with longer treatment times (20 min) resulting in the greatest decreases (*p* < 0.01). Neither AC nor SB showed a significant effect on autofluorescence in this channel, indicating limited utility for quenching Hoechst-specific autofluorescence.

In the FITC channel, the leatherleaf scaffolds exhibited strong autofluorescence in untreated controls. CS showed the greatest reduction in fluorescence intensity (46%), particularly at high concentrations with a 20 min treatment (*p* < 0.01). AC and SB also reduced autofluorescence, but their effects were less pronounced compared to CS. Among all agents, SB showed the least reduction at lower concentrations and shorter treatment times.

In the far-red (633 nm) channel, untreated controls again displayed high autofluorescence, but all three quenching agents significantly reduced intensity. CS consistently performed better than AC and SB, with the greatest reductions (52%) observed at high concentrations for 10 min treatments (*p* < 0.01). SB showed modest reductions but was less effective overall.

**Fig. 4:**
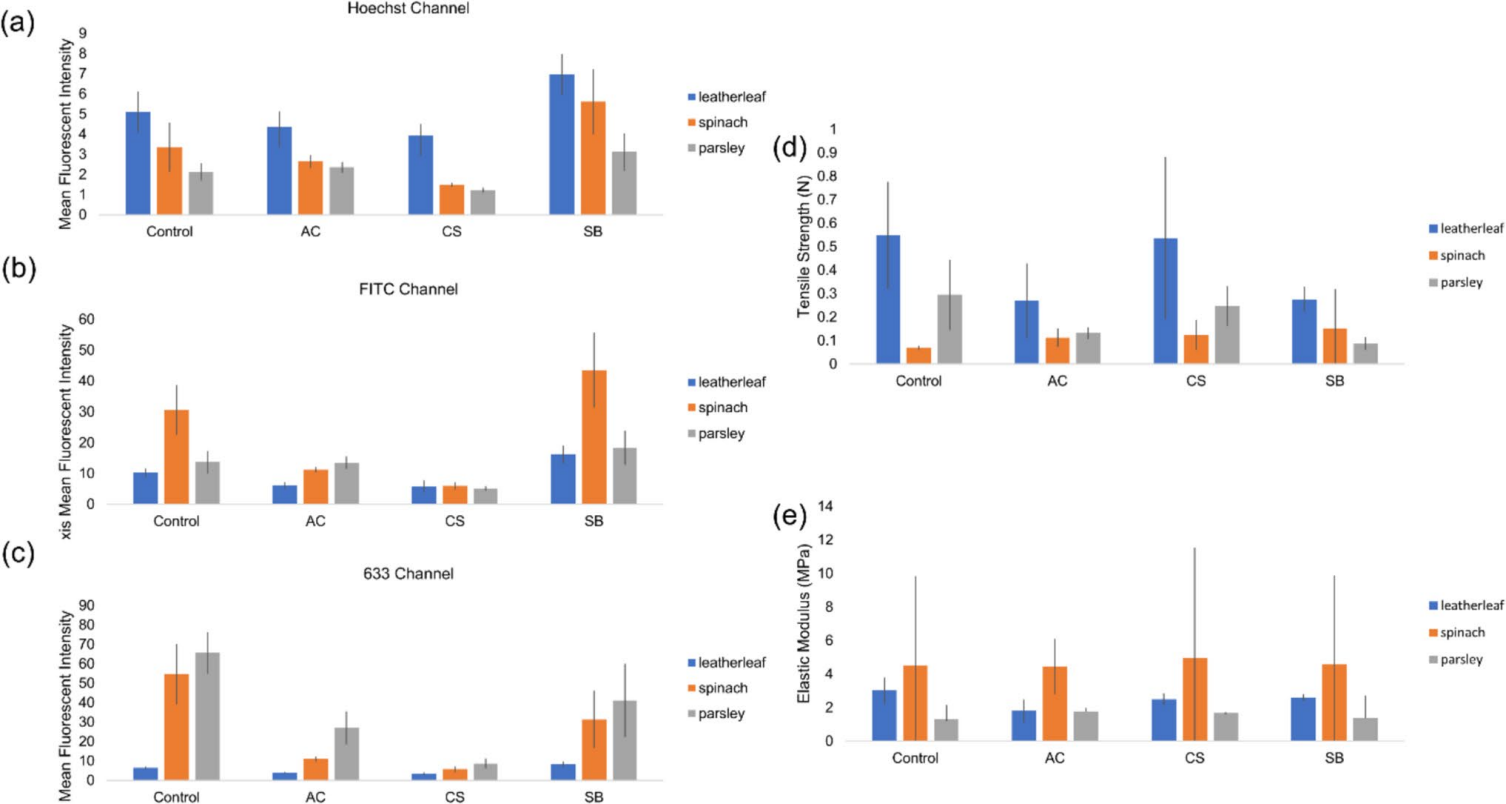
Mean fluorescence intensity in untreated and treated leatherleaf, spinach, and parsley scaffolds following 20 min quenching with copper sulfate, ammonium chloride, or sodium borohydride. **a–c** Hoechst, FITC, and 633 nm channels. Mechanical properties are shown in **d** tensile strength and **e** elastic modulus

To evaluate whether autofluorescence quenching strategies developed for leatherleaf viburnum also apply to other plant-derived scaffolds, decellularized spinach and parsley leaves were treated with CS (0.1 M), AC (0.2 M), or SB (1.0 M) for 20 min. As observed in viburnum, CS was the most effective quenching agent in both spinach and parsley (Figure 4a–c). In the Hoechst and FITC channels, CS-treated scaffolds showed significant reductions in background autofluorescence compared to untreated controls (*p* < 0.05), with lower baseline intensity in spinach than parsley. AC treatment yielded moderate reductions in the FITC and 633 nm channels but was less effective than CS. SB reduced autofluorescence to a minor extent in FITC and 633 nm channels but had little impact in the Hoechst channel.

Overall, CS emerged as the most effective quenching agent, achieving substantial reductions in autofluorescence across all tested channels. Its performance was particularly pronounced at higher concentrations and with longer treatment durations, making it the preferred agent for fluorescence imaging of plant-derived scaffolds. Its unique effectiveness in the Hoechst channel further highlights its potential for reducing scaffold autofluorescence. AC also performed well in the FITC and 633 nm channels, though less consistently than CS, while SB was the least effective quenching agent overall. The overall reduction pattern was consistent across all three scaffold types, with CS outperforming both AC and SB. Spinach scaffolds showed the highest autofluorescence in the FITC channel, while parsley exhibited more diffuse background across all channels. Figure 4 shows the reduction in autofluorescence intensity across channels and treatments in spinach and parsley scaffolds.

Mechanical testing was conducted to determine whether autofluorescence quenching affected scaffold structural properties. Decellularized leatherleaf, spinach, and parsley scaffolds were tested after treatment with CS, AC, or SB. No significant differences in tensile strength or elastic modulus were observed between treated and untreated scaffolds within each plant type. Mean values varied slightly between species, but treatment had no measurable effect on mechanical performance (Fig. 4d–e).

### Time-Dependent Quenching of Autofluorescence Across Channels

**Fig 5:**
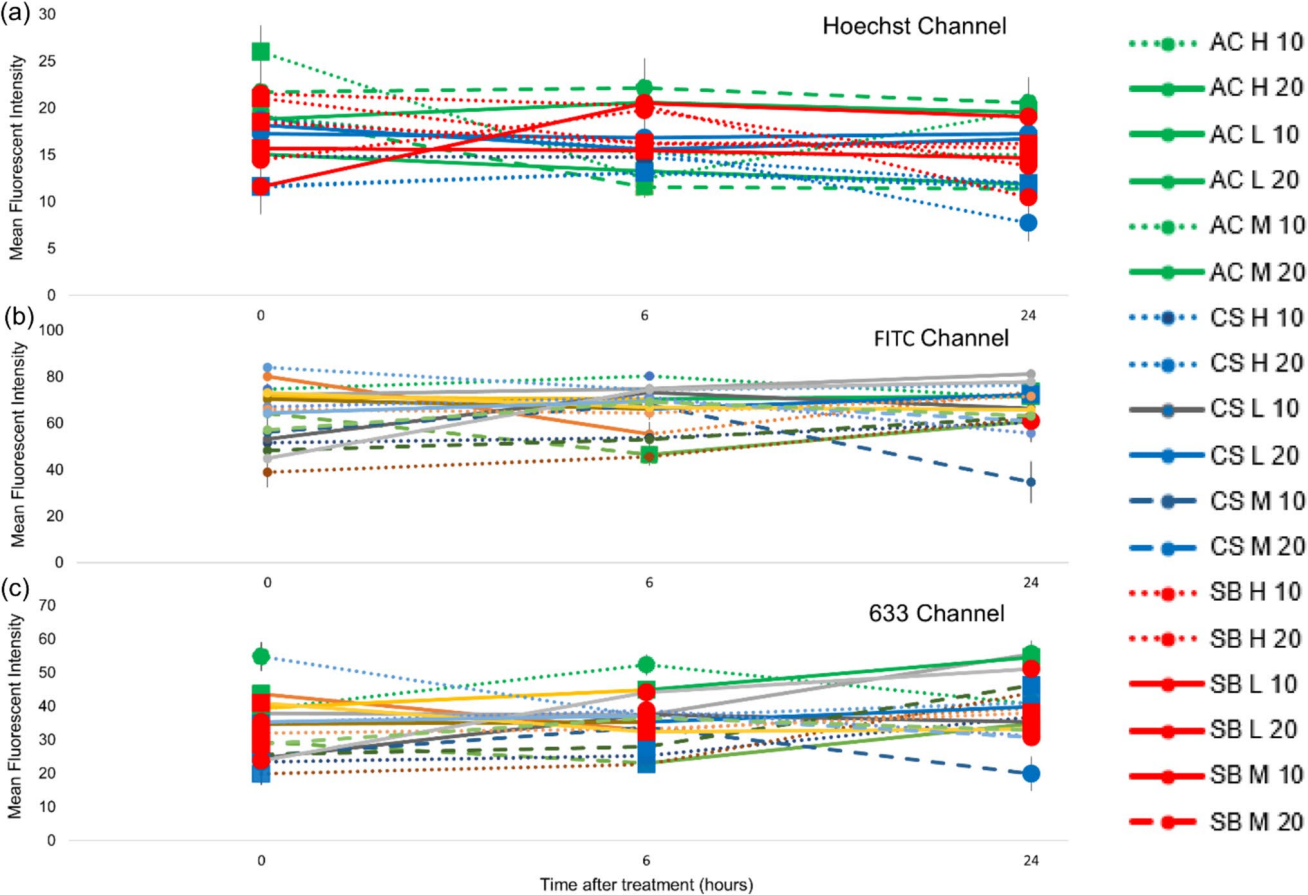
Time-dependent quenching of autofluorescence in leatherleaf over 24 h. **a–c** Hoechst, FITC, and 633 nm channels following treatment with copper sulfate, ammonium chloride, or sodium borohydride. Error bars represent standard deviation

The time-dependent effectiveness of CS, AC, and SB in quenching autofluorescence in decellularized plant scaffolds was evaluated over 24 h using Hoechst, FITC, and 633 nm channels (Fig. 5). Each quenching agent was tested at three concentrations (low, medium, and high) and two treatment times (10 and 20 min).

In the Hoechst channel (Fig. 5a), only CS demonstrated a consistent reduction in autofluorescence across all time points, with greater reductions observed at medium and high concentrations, particularly for the 20 min treatment. AC and SB showed minimal changes in fluorescence intensity, with some conditions exhibiting slight increases over time. This indicates that CS is the most effective agent for quenching autofluorescence in this spectral range.

In the FITC channel (Fig. 5b), all three quenching agents showed reductions in autofluorescence compared to untreated controls. CS exhibited the most significant and sustained reduction over time, especially at high concentrations and longer treatment durations. AC showed moderate reductions, with the effectiveness increasing with concentration and time. SB also reduced autofluorescence but was less effective compared to the other agents, particularly at lower concentrations and shorter treatment times.

In the 633 nm channel (Fig. 5c), similar trends were observed. CS again showed the most pronounced reductions in autofluorescence over time, particularly at medium and high concentrations. AC demonstrated moderate effectiveness, with reductions improving at longer treatment times and higher concentrations. SB showed the least reduction, with some conditions exhibiting increases in autofluorescence over time.

Overall, CS emerged as the most effective quenching agent across all channels, with its performance being most pronounced at higher concentrations and longer treatment durations. AC also showed efficacy, particularly in the FITC and 633 nm channels, though it was less consistent than CS. SB demonstrated limited effectiveness, with minimal reductions or even increases in autofluorescence over time under some conditions. These results highlight the superior performance of CS for reducing autofluorescence and its stability over time.

### Fluorescence Imaging of Cell-Seeded Scaffolds

**Fig 6:**
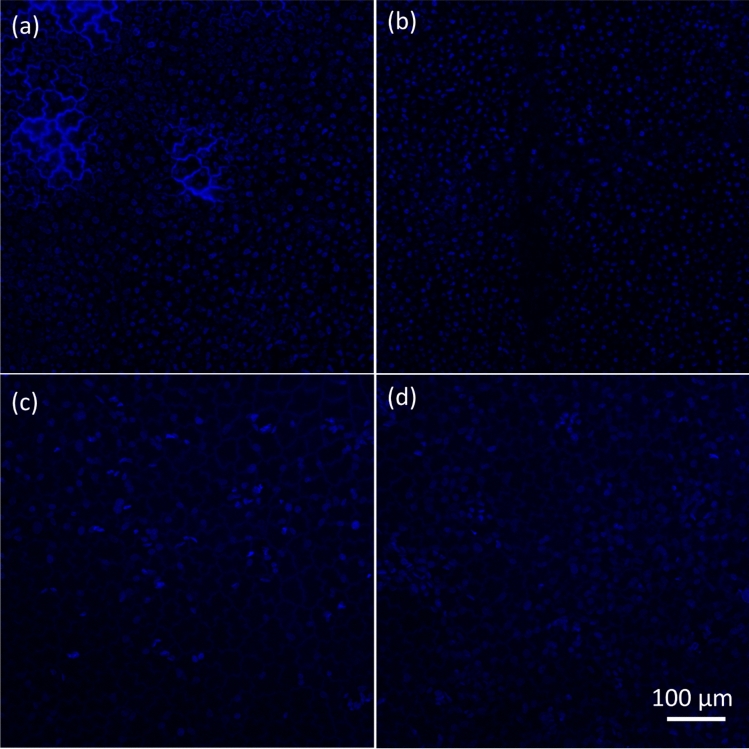
Fluorescence images of cell-seeded scaffolds. **a** EC without quenching; **b** EC with copper sulfate; **c** SMC without quenching; **d** SMC with copper sulfate. Hoechst staining highlights nuclei

Fluorescence imaging of decellularized leatherleaf viburnum scaffolds seeded with ECs and SMCs demonstrated the effectiveness of CS quenching in reducing background autofluorescence (Fig. 6). In the absence of quenching, both EC-seeded (Fig. 6a) and SMC-seeded (Fig. 6c) scaffolds exhibited strong background autofluorescence, which obscured the visibility of the seeded cells.

With CS quenching, the autofluorescence was significantly reduced, improving the clarity of cell visualization. In the EC-seeded scaffold (Fig. 6b), quenching effectively minimized background signal, allowing distinct identification of Hoechst-stained nuclei. Similarly, in the SMC-seeded scaffold (Fig. 6d), quenching reduced the autofluorescence, making the stained nuclei more prominent and the cellular distribution more discernible.

### Cell Viability After Quenching Treatment

**Fig 7.**
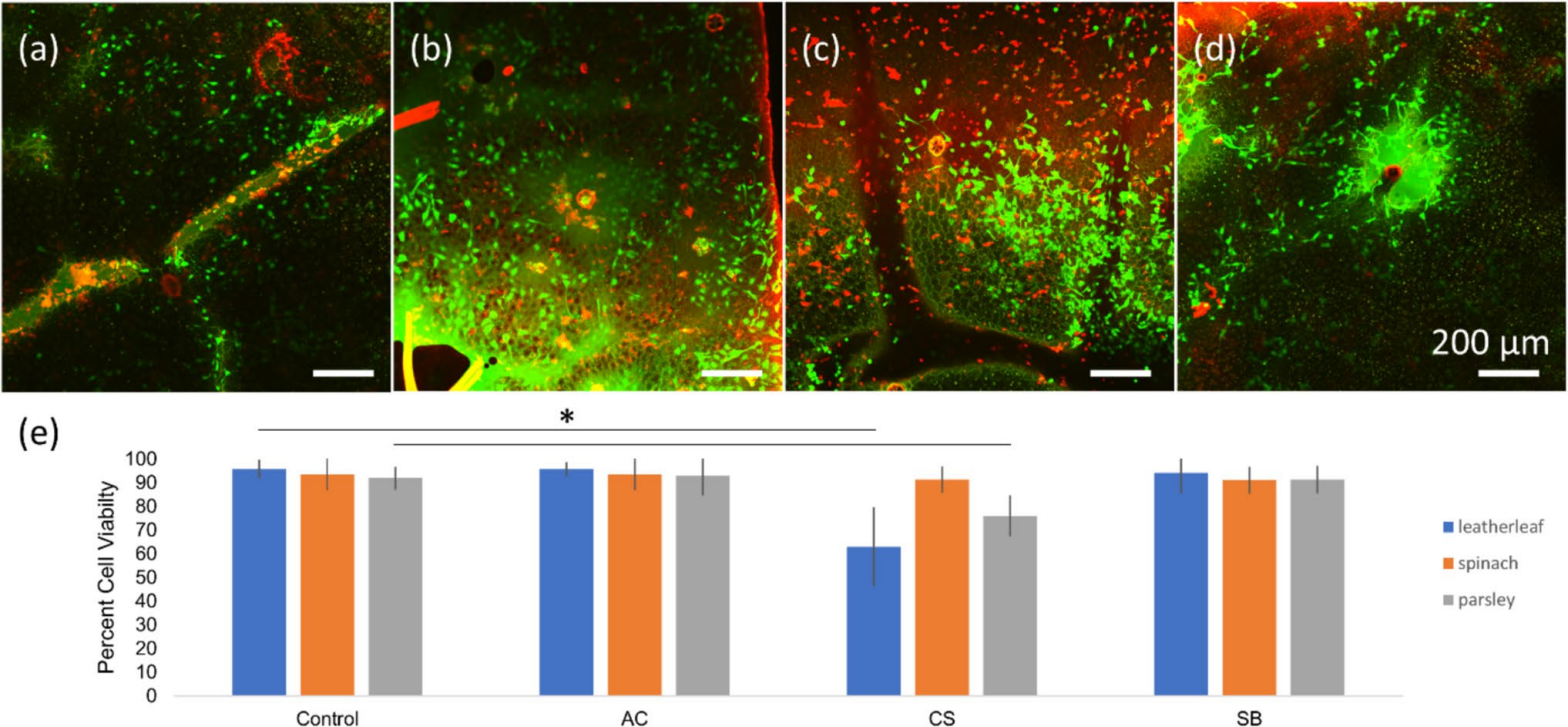
Live/dead imaging of **a** untreated and **b–d** treated leatherleaf scaffolds seeded with endothelial cells following 20 min quenching with copper sulfate, ammonium chloride, or sodium borohydride. **e** Percent cell viability

To evaluate the impact of quenching agents on scaffold biocompatibility, ECs were seeded onto treated and untreated scaffolds and assessed after 24 h using a Live/Dead assay (Fig. 7). In leatherleaf scaffolds, viability remained above 94% in control, AC, and SB conditions but declined to 63% following CS treatment. A similar trend was observed in parsley, where viability was 92% in control, AC, and SB groups and dropped to 76% with CS. In spinach scaffolds, viability remained near 93% across all conditions, including CS. These results suggest that while CS is effective for autofluorescence reduction, its effect on short-term cell viability may vary depending on scaffold type.

## Discussion

Autofluorescence from lignin and other plant compounds limits the use of fluorescence imaging in plant-derived scaffolds. This study evaluated CS, AC, and SB to address this challenge. CS reduced autofluorescence across imaging channels and time points, improving the clarity of cell visualization. By identifying a reliable quenching strategy, this study supports broader use of plant-derived scaffolds, which offer vascular-like architecture and biocompatibility in tissue engineering applications [3, 20].

Spectral scans of the decellularized leatherleaf scaffolds revealed significant autofluorescence, particularly at lower excitation wavelengths of 405 and 488 nm. This autofluorescence overlapped with the emission spectra of commonly used fluorophores, such as Hoechst and FITC, creating challenges for imaging cellular components accurately. The minimal overlap observed in the Alexa Fluor 568 (561 nm) and Alexa Fluor 647 (640 nm) channels indicates that higher wavelengths are less affected by the inherent autofluorescence of plant-derived scaffolds. This finding is consistent with previous research demonstrating that plant materials, including lignin, chlorophyll, and polyphenolic compounds, contribute to autofluorescence, particularly in the blue and green emission ranges [9]. This overlap can obscure signals from cellular markers and reinforces the need for quenching strategies or use of fluorophores in less-effected spectral ranges [3, 5]. This understanding is critical for advancing the use of plant-derived scaffolds in tissue engineering, where precise visualization of cellular structures is essential.

CS was the most effective at reducing autofluorescence across all channels, with its effect being particularly pronounced at medium and high concentrations, with longer treatment durations, and in the Hoechst channel. This efficacy can be attributed to CS’s ability to interact with autofluorescent compounds, such as phenolics and lignin derivatives, neutralizing their emission. These findings are consistent with previous reports for placenta, teratoma, and adrenal cortex [12, 21]. AC showed moderate reductions, especially in the FITC and 633 nm channels, likely due to its ability to reduce free aldehydes, a common cause of autofluorescence in fixed tissues. However, its overall performance was less consistent than CS, particularly in the Hoechst channel, where it exhibited minimal effectiveness. SB showed the least reduction in autofluorescence, and in some cases, fluorescence intensity increased over time. This inconsistency is likely due to its inability to effectively interact with plant-derived compounds responsible for autofluorescence. To assess whether these trends extended beyond leatherleaf viburnum, we tested the same quenching agents in decellularized spinach and parsley scaffolds. CS again achieved the greatest autofluorescence reduction across all channels, with AC and SB showing moderate to minimal effects, supporting the conclusion that CS quenching is broadly effective across different types of plant-derived scaffolds and can be applied to improve fluorescence imaging across structurally diverse materials.

Time-course analysis revealed that CS maintained reduced autofluorescence levels over 24 h, demonstrating superior stability compared to AC and SB. This stability is important for extended or repeated imaging, where fluctuations in autofluorescence could impact the reliability of results [22, 23]. AC exhibited moderate stability, particularly in the FITC and 633 nm channels, while SB showed the least stability, with some conditions resulting in increased autofluorescence over time. The stability of CS quenching suggests that it is well-suited for prolonged or repeated imaging protocols, reducing the need for re-treatment and supporting consistent imaging conditions in scaffold-based tissue engineering studies.

CS quenching significantly improved the visualization of Hoechst-stained nuclei in scaffolds seeded with ECs and SMCs. In unquenched scaffolds, high background autofluorescence obscured cellular signals, making it challenging to assess cell morphology and distribution accurately. With CS treatment, autofluorescence was substantially reduced, resulting in enhanced contrast between the scaffold and the seeded cells. These findings are particularly important for studies involving cell-seeded scaffolds, where reducing background autofluorescence with CS enables more precise imaging of cellular morphology and supports reliable assessment of scaffold biocompatibility and function in fluorescence-based assays.

While CS effectively suppressed autofluorescence, its impact on cell viability differed by scaffold. ECs tolerated CS-treated spinach scaffolds well, but viability declined in leatherleaf and parsley, suggesting scaffold-specific retention of copper ions or surface interactions may contribute to cytotoxicity. Prior studies using CS in fixed tissues reported minimal adverse effects [13, 14], but studies in live cells confirm dose- and time-dependent toxicity [24, 25]. This suggests that residual copper may impair viability if not thoroughly rinsed, particularly in lignin-rich scaffolds. For live-cell imaging, AC or SB may be better suited, though SB is known to be cytotoxic at high doses [26]. CS is best reserved for post-fixation quenching, where it remains the most effective option for suppressing autofluorescence without compromising imaging quality.

In addition to imaging performance, it was important to assess whether quenching treatments compromised the physical integrity of the scaffolds. Mechanical testing demonstrated that neither tensile strength nor elastic modulus was significantly affected by any of the quenching agents across all three plant types. This suggests that the quenching protocols used here preserve the bulk mechanical properties of the scaffolds, supporting their continued use in applications where mechanical function is critical, such as vascular graft development.

While CS demonstrated strong performance as a quenching agent, this study was limited to three agents. Future research could explore additional quenching agents or alternative methods to optimize autofluorescence reduction further. Expanding this study to include a broader range of cell types and scaffold designs, as well as dynamic culture conditions, could provide further insights into the versatility and limitations of CS quenching. The inclusion of spinach and parsley strengthens the practical relevance of this study, as both are widely used plant scaffolds in tissue engineering.

This study highlights the effectiveness of CS for reducing autofluorescence in decellularized leatherleaf viburnum, spinach, and parsley scaffolds. By significantly reducing background signals and enhancing cellular visualization, CS addresses a key challenge in fluorescence imaging of plant-derived scaffolds. These findings contribute to the growing body of research on plant-based scaffolds and provide practical guidance for improving imaging quality in scaffold-based tissue engineering studies. The superior performance and stability of CS demonstrate its potential as a valuable tool for researchers in the field.
